# Expert-Moderated Peer-to-Peer Online Support Group for People With Knee Osteoarthritis: Mixed Methods Randomized Controlled Pilot and Feasibility Study

**DOI:** 10.2196/32627

**Published:** 2022-01-17

**Authors:** Thorlene Egerton, Belinda J Lawford, Penny K Campbell, Melanie L Plinsinga, Libby Spiers, David A Mackenzie, Bridget Graham, Kathryn Mills, Jillian Eyles, Gabrielle Knox, Ben Metcalf, Liam R Maclachlan, Manuela Besomi, Chris Dickson, Charles Abraham, Bill Vicenzino, Paul W Hodges, David J Hunter, Kim L Bennell

**Affiliations:** 1 Centre for Health, Exercise & Sports Medicine The University of Melbourne Melbourne Australia; 2 Physiotherapy Department The University of Melbourne Melbourne Australia; 3 Menzies Health Institute Queensland Griffith University Brisbane Australia; 4 Discipline of Physiotherapy Macquarie University Sydney Australia; 5 Institute of Bone and Joint Research Kolling Institute of Medical Research, Faculty of Medicine and Health The University of Sydney Sydney Australia; 6 Kenneth G. Jamieson Department of Neurosurgery Royal Brisbane and Women’s Hospital Brisbane Australia; 7 School of Health and Rehabilitation Sciences The University of Queensland Brisbane Australia; 8 Department of Integrative Medicine and Supportive Care Chris O’Brien Lifehouse Sydney Australia; 9 School of Psychology Deakin University Geelong Australia

**Keywords:** support group, online support group, knee, osteoarthritis, arthritis, online forums, patient education, self-efficacy, health literacy, self-management, qualitative

## Abstract

**Background:**

Osteoarthritis (OA) is a major problem globally. First-line management comprises education and self-management strategies. Online support groups may be a low-cost method of facilitating self-management.

**Objective:**

The aim of this randomized controlled pilot study is to evaluate the feasibility of the study design and implementation of an evidence-informed, expert-moderated, peer-to-peer online support group (My Knee Community) for people with knee OA. The impacts on psychological determinants of self-management, selected self-management behaviors, and health outcomes were secondary investigations.

**Methods:**

This mixed methods study evaluated study feasibility (participant recruitment, retention, and costs), experimental intervention feasibility (acceptability and fidelity to the proposed design, including perceived benefit, satisfaction, and member engagement), psychological determinants (eg, self-efficacy and social support), behavioral measures, health outcomes, and harms. Of a total of 186, 63 (33.9%) participants (41/63, 65% experimental and 22/63, 35% control) with self-reported knee OA were recruited from 186 volunteers. Experimental group participants were provided membership to My Knee Community, which already had existing nonstudy members, and were recommended a web-based education resource (My Joint Pain). The control group received the My Joint Pain website recommendation only. Participants were not blinded to their group allocation or the study interventions. Participant-reported data were collected remotely using web-based questionnaires. A total of 10 experimental group participants also participated in semistructured interviews. The transcribed interview data and all forum posts by the study participants were thematically analyzed.

**Results:**

Study feasibility was supported by acceptable levels of retention; however, there were low levels of engagement with the support group by participants: 15% (6/41) of participants did not log in at all; the median number of times visited was 4 times per participant; only 29% (12/41) of participants posted, and there were relatively low levels of activity overall on the forum. This affected the results for satisfaction (overall mean 5.9/10, SD 2.7) and perceived benefit (17/31, 55%: *yes*). There were no differences among groups for quantitative outcomes. The themes discussed in the interviews were *connections and support*, *information and advice*, and *barriers and facilitators*. Qualitative data suggest that there is potential for people to derive benefit from connecting with others with knee OA by receiving support and assisting with unmet informational needs.

**Conclusions:**

Although a large-scale study is feasible, the intervention implementation was considered unsatisfactory because of low levels of activity and engagement by members. We recommend that expectations about the support group need to be made clear from the outset. Additionally, the platform design needs to be more engaging and rewarding, and membership should only be offered to people willing to share their personal stories and who are interested in learning from the experiences of others.

**Trial Registration:**

Australian New Zealand Clinical Trials Registry ACTRN12619001230145; http://anzctr.org.au/Trial/Registration/TrialReview.aspx?id=377958

## Introduction

### Background

Osteoarthritis (OA) is a major cause of pain, disability, and health service use globally [1]. Prevalence and burden are predicted to increase because of population aging and rising obesity rates [1,2]. Recommended management emphasizes nonsurgical, nondrug treatment, including physical activity and weight loss [3-6], delivered through education and self-management support. Current management of the condition in Australia and worldwide has been found to be inconsistent with recommended practice [7-9]. Given these deficiencies with current care and the scale of the problem, additional, inexpensive, and scalable resources or services, which can help meet care needs and close care gaps, need to be explored.

A community survey by Arthritis Australia found that people who fare worse because of their joint pain are those who perceive they have received poorer care through lack of information and access to help rather than those who have worse disease severity or longer disease duration [10]. More recently, contextual factors, specifically *support and relationships* were found to be key influencers of how a person manages their OA [11]. Having supportive friends, role models, and opportunities to share experiences with others were all found to help people maintain independence. They also helped people stay active and adopt effective lifestyle self-management behaviors. Studies also suggest that support networks can be a source of health information [12]. Thus, improving life participation and satisfaction for people living with knee OA may be aided through having access to a social network that provides accurate information and greater feelings of support and connectedness, while also meeting individual needs.

Peer-to-peer online support groups (OSG) can be defined as “any virtual social space where people come together to get and give information or support, to learn, or to find company” [13]. OSGs can potentially be a low-cost method of providing peer support and information [12,14]. Groups can also provide effective behavior change interventions [15]. They offer several advantages over face-to-face support groups in terms of accessibility, time, and financial cost to participants; running and maintenance costs; and the asynchronous nature of engagement [12,14,16]. In addition, participants may find it easier to disclose personal or sensitive information in a web-based environment rather than in person. Online group facilitation, as with web-based consulting, may be an increasingly feasible way of bringing patients with similar health problems together [17]. Studies on OSGs for other conditions support their effectiveness for improving emotional well-being, self-efficacy, and feelings of support [12,18,19]. Little is known about the effectiveness of OSGs for chronic painful musculoskeletal disorders or knee OA. Research is also needed to determine the features and functions needed to optimize engagement and hence effectiveness and to understand the mechanisms by which positive effects are achieved [12]. Existing evidence suggests that important features to include are perceived similarity among support group members (eg, having the same disease), credibility of information, access to experts or trained peer facilitators, and enjoyment [14].

### Objectives

We conducted a mixed methods randomized controlled pilot and feasibility study with people with knee OA who were provided with peer-to-peer support via an expert-moderated OSG and an OA information website. The comparison group were only provided with the OA information website. The primary objective is to determine the feasibility of delivering the OSG intervention in a trial setting. Feasibility was explored in terms of the study methods (participant *recruitment and retention* and *costs*) and the experimental intervention (perceived *benefit*, *satisfaction,* and *engagement* with the OSG). Secondary objectives include the impacts of participation in an OSG on psychological determinants of self-management and lifestyle behavior change, behaviors, and health outcomes compared with those of participants who only received web-based information.

## Methods

### Overview

The study was conducted in accordance with the published trial registration (ACTRN12619001230145), the conditions of ethics committee approval (University of Melbourne Human Research Ethics approval number: 1853275.4), and the Note for Guidance on Good Clinical Practice (CPMP/ICH-135/95). This report follows the guidance of the Consolidated Standards of Reporting Trials (CONSORT) extension for randomized pilot and feasibility trials [20], Consolidated Standards of Reporting Trials-eHealth [21], and Consolidated Criteria for Reporting Qualitative Research [22] for the qualitative research component [22]. Study participants provided verbal and digital informed consent.

### Study Design

The study was a 2-arm, pragmatic randomized parallel-groups design pilot and feasibility study with mixed methods analysis.

### Participants

Volunteers from Australia, who had self-reported clinically diagnosed knee OA were recruited from research databases, Facebook, and an advertisement on the Arthritis Australia website. Participants were eligible for the study if they (1) were aged >45 years, (2) self-reported having been diagnosed with knee OA by a physician or met the clinical criteria for knee OA (activity-related knee pain on most days, experienced pain for at least three months, and no morning joint-related stiffness lasting >30 minutes) [23], (3) could access and were willing to use the internet at least once a week, (4) were prepared to engage in an OSG if randomized to that group, (5) able to commit to completing baseline and follow-up questionnaires, and (6) able to give informed consent. Potential participants were excluded if they (1) had undergone previous knee replacement on their painful knee or were on the waiting list for knee surgery, (2) self-reported a diagnosis of rheumatoid arthritis or other inflammatory arthritis, (3) were currently participating in an arthritis support group, or (4) had another serious medical condition or upcoming medical procedures that in the opinion of the research staff would preclude participation. Screening was done in 2 stages via a web-based survey (Qualtrics) and a phone call (LS and GK).

### Study Procedures

Participant recruitment, the provision of consent, interventions, and assessments were all performed on the web. Following informed consent, participants completed the baseline questionnaire on a secure web-based platform designed to support data capture for research studies [24,25]—REDCap (Research Electronic Data Capture; Vanderbilt University)—hosted at the University of Melbourne. Full disclosure was provided, so participants were not blinded to the different types of web-based resources being compared in this study. As all impact assessments used self-report, the participants were the assessors and therefore were not blinded to treatment group allocation or intervention provided to both groups. A researcher not involved in generation or implementation of the randomization schedule revealed group allocation via REDCap. Participants were then informed of their group allocation and how to access the relevant websites. For the OSG group, this is reflective of real life where participants were always aware of the format with which they received information and education or peer support and had preconceived views on the benefit and relative effectiveness of these options.

### Intervention and Control Conditions

Experimental group participants were provided membership to an OSG for people with knee OA (My Knee Community) and recommended a web-based patient education resource (My Joint Pain [26]) [27]. Control group participants received the My Joint Pain website recommendation only.

My Knee Community was a newly established, expert-moderated, peer-to-peer OSG hosted with Discourse web-based platform (Civilized Discourse Construction Kit, Inc). It provides an online discussion forum organized into categories (eg, *Living with knee osteoarthritis*) and threads (eg, *Cold weather and joint pain*). Before study commencement, the forum was reviewed and tested by approximately 15 stakeholders, including 3 individuals with knee OA. My Knee Community was then opened for membership (August 14, 2019) and promoted on Facebook; the Arthritis Australia website; the Centre for Health, Exercise and Sports Medicine webpage, and internet search engines (unpaid). It was a closed group, and interested people were screened via a web-based survey (Qualtrics) for self-reported knee OA and Australian location before being given member access. When pilot study recruitment commenced (December 11, 2019), there were 123 members and 84 posts, of which 12 were moderator posts.

My Knee Community members could add posts to threads or create new threads. The OSG was monitored (daily) and moderated (approximately weekly) by *experts*, who were health care professionals (mostly physiotherapists) with knowledge of evidence-based management of knee OA and a belief that people with knee OA can learn self-management skills. The moderator’s roles included (1) removing any offensive posts or product advertisements, (2) posting information, such as new research findings or links to recommended knee OA resources, and (3) contributing to discussions by responding to questions and comments if they needed or requested a response from a health care professional.

All members were permitted to view and post as much or as little as they wanted, but members who were study participants were asked to log on to the My Knee Community at least once. Thus, one or more visits to the OSG constituted adherence to the experimental intervention protocol. All randomized participants’ data were included in the analysis unless they were lost to follow-up. After the final follow-up questionnaire, experimental group participants could remain members of the My Knee Community and control group participants were offered membership.

My Joint Pain [26] is a freely available website managed by Arthritis Australia and developed for Australian users with joint pain. It provides information via factsheets, videos, and other tools, such as risk assessments. Some resources require signing up for access.

### Sample Size

The sample size of 60 for this study was based on recommended sample sizes for feasibility studies [28,29] and the recommended sample size for pilot studies using continuous variables and small (0.2) effect sizes [30]. The randomization schedule was computer generated in advance by a statistician not involved in the study according to a 2:1 ratio using random permuted blocks of varying sizes (4-10). Allocation was concealed in a password-protected software (REDCap). An unequal sample size between experimental and control groups was used because most of the research questions were related to the experimental group data. This strategy enabled more data to be collected from experimental group participants, particularly for the qualitative evaluations, for a smaller overall sample size but without compromising the ability to answer all feasibility questions. Finally, a target of 40 experimental group participants was estimated to be an adequate sample size for the qualitative components.

### Quantitative Evaluation—Feasibility of Study Methods and Experimental Intervention

Data for the quantitative feasibility evaluation (ie, participant recruitment, participant retention, costs, perceived benefit, satisfaction, and engagement with the OSG) were collected after 3 months using a questionnaire or at the end of the trial (ie, for data analytics) and are reported descriptively (Multimedia Appendix 1). Participants completed baseline and follow-up questionnaires (3 months after randomization) for the impact evaluation. Questionnaires measured psychological determinants (motivation, activation, self-efficacy, health education impact, health literacy, coping, social support, and fear of movement), self-management behaviors (physical activity, exercise, and weight loss), health outcomes (quality of life, pain, fatigue, function, sleep, mood, and global rating of change), and harms (related adverse events).

### Quantitative Evaluation—Psychological Determinants

**Figure 1:**
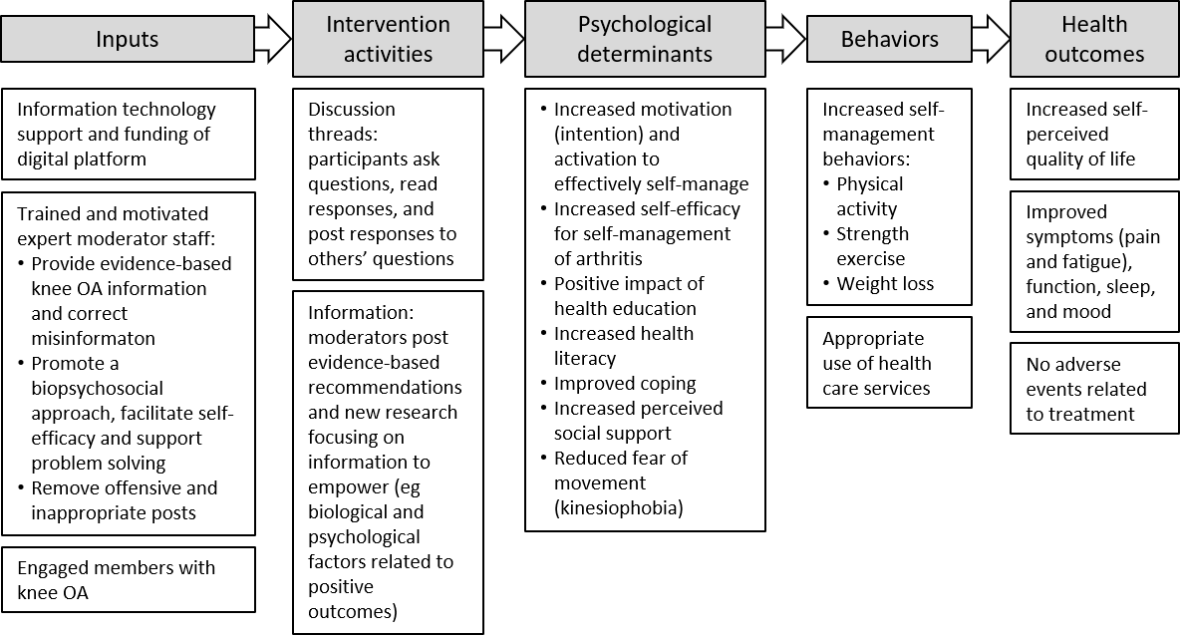
Program logic describing the study intervention. OA: osteoarthritis.

Psychological determinants are constructs that were hypothesized to be important in the causal pathway through which an OSG can achieve increased uptake of effective self-management behaviors and improved health outcomes (Figure 1). Motivation was measured by asking participants to rate their level of agreement with statements about motivation to be more active or exercise or lose weight on 11-point numerical rating scales (NRS) [31]. Activation was measured using the Patient Activation Measure [32], and coping was measured using the Brief Coping Strategy Questionnaire [33], both of which have been shown to be valid measures in knee OA populations [33,34]. Self-management skills were measured using the Health Education Impact Questionnaire, which has high construct validity and reliability in a sample of individuals with chronic conditions including arthritis [35]. Health literacy was measured with the Health Literacy Questionnaire, which shows good reliability and validity in a broad Australian community sample [36]. Social support was measured using the Duke-University of North Carolina Functional Social Support Questionnaire [37], which has moderate reliability and validity in community-dwelling older Australians [38]. Fear of movement (kinesiophobia) was measured using the Brief Fear of Movement Scale for Osteoarthritis [39] and self-efficacy, using the Arthritis Self-Efficacy Scale [40]. Both measures were developed for individuals with knee and hip OA and demonstrate acceptable validity and reliability [39,40].

Physical activity during the past week was measured using the Incidental and Planned Exercise Questionnaire as well as by asking participants “How many days in the past week did you do 30 minutes of moderate intensity physical activity?” The Incidental and Planned Exercise Questionnaire (past week) has good validity and reliability in a sample of older adults [41]. Strength exercise was captured similarly, with participants answering “How many days in the past week did you do leg strengthening exercises?” Participants were also asked “If you need to lose weight, how much effort are you currently making?” on a 11-point NRS.

Quality of life was measured using the Assessment of Quality of Life instrument [42], which has good validity in a sample of Australians with hip and knee joint disease [43]. Pain, fatigue, and sleep were measured by asking participants to rate each on a 11-point NRS. Measurement of pain in this way is recommended for OA clinical trials by the Osteoarthritis Research Society International [44]. There is evidence of validity for this measurement of fatigue [45] and sleep [46] albeit not yet in individuals with OA. Physical function was measured using the Western Ontario and McMaster Universities Osteoarthritis Index physical function subscale [47] and global change by a 7-point Likert scale asking about o*verall change in knee condition since commencing in the study*. Both measures are recommended for use in OA clinical trials [44], and the Western Ontario and McMaster Universities Osteoarthritis Index is valid, reliable, and responsive in OA populations [47]. Finally, mood was measured using the Patient Health Questionnaire-9, which has high reliability and validity in arthritis populations [48].

Adverse events were collected by asking participants “Have you had any new health problems or symptoms, or have any of your existing health conditions or symptoms worsened since you started in the study?” Further details of the impact evaluation are provided in Multimedia Appendix 2.

Data are reported descriptively by treatment group assignment. Between-group differences in change were statistically analyzed by linear regression modeling with follow-up value as the dependent variable and baseline value and group allocation as independent variables. This was to help identify outcomes that may favor the intervention group rather than to determine effectiveness. Statistical analysis was conducted using SPSS software (version 26; IBM Corp). If a participant chose not to engage at all with the OSG, they were considered a protocol violator; however, provided that they completed follow-up questionnaires, their data remained in the analysis, and all completers were analyzed according to their allocated group.

### Qualitative Evaluations

In total, 2 qualitative evaluations were nested within the study. The first qualitative evaluation used semistructured interviews and reflexive thematic analysis [49] to explore the perspectives and experiences of participants in the My Knee Community OSG. All experimental group participants were invited to participate in telephone interviews after completing their 3-month questionnaire. All participants who agreed were interviewed. The final sample was therefore not dictated by data saturation. Interviews were conducted by an experienced interviewer (PKC, who is a research trial coordinator, woman with previous experience in OA research, trained in qualitative interview methods, and a part of the study team but not previously known to participants or involved in My Knee Community). The topics discussed included their experiences, perceived impacts, and perceived barriers and enablers to engagement. The interview guide (Multimedia Appendix 3) was developed directly from these study aims. Interviews were audio recorded and transcribed. Analysis of transcribed interviews was based on an inductive thematic approach informed by grounded theory [50]. This method involves the generation of hypotheses and theories from data through cumulative coding [51]. Transcripts were read separately by BJL and PKC after transcription then coded to identify topics and patterns of ideas within the data. Both researchers independently organized codes into categories before meeting to discuss ideas. To ensure credibility and confirmability of the data, another researcher (MP) read all transcripts before meeting with BJL and PKC to review initial themes and subthemes. A fourth researcher (TE) provided additional input and validation of final themes and subthemes. All analytical steps were performed using word processing software.

The second qualitative evaluation was a content analysis [52] of posts by study participants to understand how the OSG for people with knee OA was used by people living with the condition. This involved 2 researchers (BJL and TE) independently reading through all posts and coding the data to identify topics and initial patterns of ideas, which were then grouped into categories. Categories were given descriptive labels and reported as themes.

## Results

### Overview

**Figure 2:**
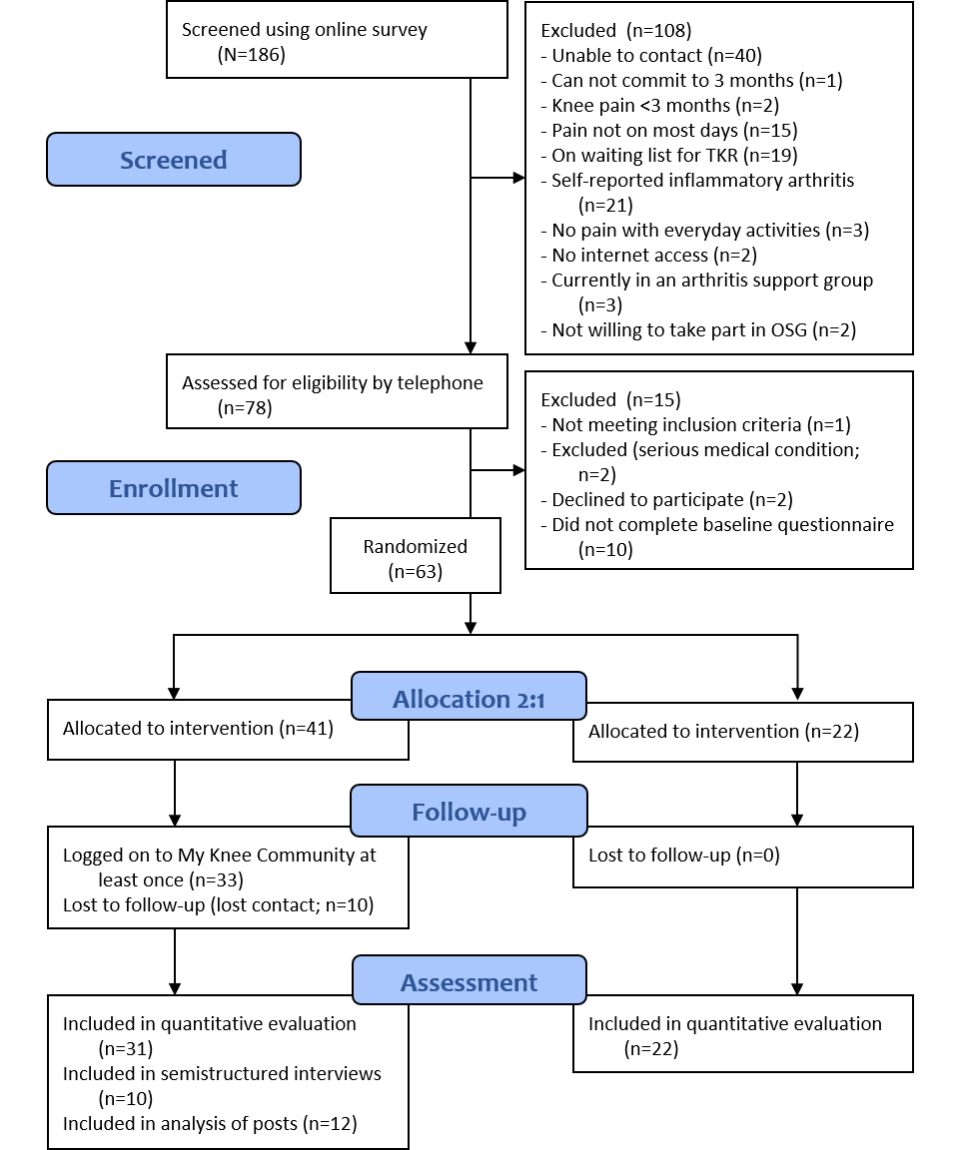
Flow of participants through the study [18]. OSG: online support group; TKR: total knee replacement.

A total of 186 volunteers were screened on the web, with 63 (33.9%) participants recruited (41/63, 65% participants allocated to OSG and 22/63, 35% participants, to control) between December 2019 and May 2020 (Figure 2). Participants (mean age 62.6 years, SD 11.2 years; 52/63, 83% were women; Table 1) included people living in different locations within Australia, including approximately 20% (12/63) in outer regional or remote areas. They had a range of educational levels and approximately half were in paid work. Duration of symptoms varied in participants from <1 year (6/63, 10%) to >10 years (14/63, 22%). A total of 5% (3/63) of participants had previously attended a self-management program, but none had previously participated in a support group. A total of 100% (22/22) of control participants and 76% (31/41) of OSG participants completed the follow-up questionnaire; 24% (10/41) of OSG participants were lost to follow-up (Figure 2).

**Table 1:** Study participant characteristics (N=63).

Characteristics			Control group participants (n=22)		Online support group participants (n=41)
Age (years), mean (SD)			66.4 (7.6)		60.5 (12.4)
**Sex, n (%)**					
	Female	18 (82)		34 (83)	
**Region, n (%)**					
	Major city	8 (36)		24 (59)	
	Inner regional	10 (46)		9 (22)	
	Outer regional	3 (14)		8 (20)	
	Remote	0 (0)		0 (0)	
	Very remote	1 (5)		0 (0)	
**Highest level of education, n (%)**					
	Some secondary or high school	1 (5)		4 (10)	
	Completed secondary or high school	0 (0)		7 (17)	
	Completed some further study (eg, Technical and Further Education)	9 (41)		16 (39)	
	Completed university: bachelor’s degree	9 (41)		8 (20)	
	Completed university: master’s degree	3 (14)		6 (15)	
**Current employment status, n (%)**					
	Work: full time (paid)	2 (9)		8 (20)	
	Work: casual (paid)	1 (5)		1 (2)	
	Work: part time (paid)	7 (32)		10 (24)	
	Unable to work because of health reasons	1 (5)		4 (10)	
	Retired (not because of health reasons)	11 (50)		15 (37)	
	Unemployed or not employed (eg, caring)	0 (0)		3 (7)	
**Duration of knee pain symptoms, n (%)**					
	<1 year	2 (9)		4 (10)	
	1 or 2 years	5 (23)		4 (10)	
	3-5 years	6 (27)		11 (27)	
	5-10 years	5 (23)		12 (29)	
	>10 years	4 (18)		10 (24)	
**When first consulted a physician for knee pain, n (%)**					
	<1 year ago	2 (9)		2 (5)	
	1 or 2 years ago	3 (14)		5 (12)	
	3-5 year ago	6 (27)		4 (10)	
	5-10 years ago	5 (23)		12 (29)	
	>10 years ago	2 (9)		8 (20)	
**Previous participation, n (%)**					
	Any support group	0 (0)		0 (0)	
	Any online support group	0 (0)		0 (0)	
	Any other self-management program	2 (9)		1 (2)	
**Problems around other joints, n (%)**					
	None	0 (0)		0 (0)	
	Head	0 (0)		2 (5)	
	Neck	11 (50)		14 (34)	
	Back	10 (46)		22 (54)	
	Hip or hips	5 (23)		22 (54)	
	Foot, ankle, or ankles	11 (50)		17 (42)	
	Shoulder	6 (27)		6 (15)	
	Elbow or elbows	10 (46)		12 (29)	
	Hand, hands, wrist, or wrists	0 (0)		0 (0)	

### Intervention Activities (Experimental Intervention Feasibility)

A total of 85% (35/41) of OSG study participants accessed My Knee Community at least once and therefore were considered adherent to the study protocol (Table 2). A total of 15% (6/41) of participants did not log in at all and approximately half logged in <3 times. Mean (SD) number of times visited per participant was 8.2 (SD 12.9; median 4, range 0-75). Only 29% (12/41) of participants posted, with the median number of posts per participant being 2 (range 1-5) for those who posted. During the study period (December 12, 2019, to August 31, 2020), there were 77 new members in the OSG (including the 35 new members who were study participants). Study participant engagement during the study period was consistent with the activity by members overall, that is, from the 200 total My Knee Community membership by the end of the study period, there were 922 total user visits during the study period (mean 4.6 per member) and 100 posts, with one-third being moderator posts and one-fourth being made by study participants (mean 0.3 post per member and mean 2.7 new posts per week). The topic with most reads and most replies was *Will exercise help my osteoarthriti*s*?*, followed by w*eight loss and reduction in pain: the evidence* and then s*upplements to reduce inflammation*. The post with most likes was as follows:

For me it’s simple walking. I’ve gradually built up so now I can do 10-12km each walk. I walk at a pace of 6km/hr. I love it. In fact, I find my knee is more painful if I don’t walk! The other benefits I’ve had is that I am now stronger in my legs and this has made it easier to do some movements that I found very painful before, like walking downstairs. The walking is free, and I also get to be outside which helps my mood.

Comparing self-reported with software analytics data, participants either underestimated their number of log-ins or those who visited the OSG the most did not complete the follow-up questionnaire (Table 2). Perceived benefit was lower for the OSG intervention than for the information website, with 55% (17/31) of the OSG participants saying that they benefited from the My Knee Community but approximately three-fourth of the control group saying that they benefited from My Joint Pain (Table 2). Satisfaction was moderate, with an average rating for overall satisfaction with the My Knee Community of 5.9/10 (SD 2.7) and satisfaction with specific aspects ranging from an average of 4.2/10 (SD 3.0) for *relationships developed with other participants* to 6.4/10 (SD 3.1) for *input from expert moderator* (Table 2).

For the qualitative content analysis of posts, 29 posts were made during the study period by 12 study participants who were My Knee Community members. From these, 4 themes were identified. The first theme included describing their successes (n=6 posts). These narratives included positive descriptions of management methods they use:

In the past 18 months I have lost 18 kgs just through a few minor changes. The difference in my knees is remarkable, as well as my feet. I now am more active, and my doctor believes that I have avoided surgery. My knee pain is bearable, the only time it flares up is when I am inactive.

MKC9

The second theme included describing their struggle (n=6 posts). These posts described the difficulties people had coping because of their knee problem:

I am in Melbourne and feeling the stress of not being able to really go anywhere. I’m not sleeping well, eating erratically and any exercise just feels like it’s all ‘too hard’ atm.

MKC10

The third theme included what they do (n=9 posts), which included neutral narratives of management methods they use or their experiences of living with the condition:

I experience the same with cooler weather and high humidity. Rather than take anything, I use Voltaren gel and the TENS machine, not necessarily in that order 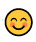.

MKC6

The fourth theme included appreciating something (n=7 posts). These generally short posts expressed thanks for a resource that was mentioned on the community forum:

Great video guys. It made a lot of sense and motivated me.

MKC7

Only 1 of the 29 posts analyzed asked for advice:

Has anyone tried Synvisc injections into their knee?

MKC9

**Table 2:** Experimental intervention feasibility.

Experimental intervention (online support group) feasibility						Participants
**Self-reported measures (n=31)**						
	**Perceived benefit (My Joint Pain), n (%)**					
		Do you think you benefited from using the information website? Yes		23 (74)		
	**Perceived benefit (My Knee Community), n (%)**					
		Do you think you benefited from using the online support group? Yes		17 (55)		
	**How satisfied were you with the My Knee Community?** **(0-10 numerical rating scale: not at all satisfied to completely satisfied), mean (SD)**					
		Overall		5.9 (2.7)		
		Quality of advice and information		4.9 (3.2)		
		Amount of information		6.3 (3.0)		
		Ease of use		5.9 (3.2)		
		Relationships developed with other participants		4.2 (3.0)		
		Input from expert moderator		6.4 (3.1)		
	**Engagement**					
		**How many times did you visit My Knee Community over the past 3 months? n (%)**				
			Never	3 (10)		
			1-2 times	10 (32)		
			3-5 times	14 (45)		
			6-10 times	3 (10)		
			>10 times	1 (3)		
		**How often did you read posts? n (%)**				
			Never or rarely	7 (23)		
			Once every 2 or 3 weeks	22 (71)		
			Once or twice per week	2 (6)		
			>Twice per week	0 (0)		
		**How often did you post on the discussion board? n (%)**				
			Never or rarely	28 (90)		
			Once every 2 or 3 weeks	3 (10)		
			Once or twice per week	0 (0)		
			>Twice per week	0 (0)		
**Software analysis (n=41)**						
	**Number of times visited, n (%)**					
		Never		6 (15)		
		1-2 times		8 (20)		
		3-5 times		13 (32)		
		6-10 times		3 (7)		
		>10 times		11 (27)		
	Number of times visited, median times visited per participant (range)				4 (1-75)	
	Number of topics viewed, median topics per participant (range)				7 (0-35)	
	Number of posts read, median posts read per participant (range)				38 (0-164)	
	Number of posts created, median posts created per participant (range)				0 (0-5)	
	**Number of participants categorized,** **n (%)**					
		No participation^a^		6 (15)		
		Lurkers^b^		23 (56)		
		Posters^c^		12 (29)		

^a^Did not log in to My Knee Community at all.

^b^Logged in but did not post.

^c^Logged in and posted at least once.

### Study Feasibility

A total of 94% (59/63) of participants were recruited from paid advertisements (Facebook and Instagram), with the remaining 4 recruited from free advertisements (university webpage, participant database, and word of mouth). Total cost of recruiting was Aus $1086.20 (US $772), which equates to Aus $17.24 (US $12) per participant recruited. Recruitment rate was 7.9 participants per week. A total of 84% (53/63) of participants completed the 3-month questionnaire. Retention was 100% (22/22) for the control participants and 76% (31/41) for the OSG participants. A total of 33 posts were made by the moderator during the study period, which equated to approximately 10 hours work over 8.5 months. Moderator posts were mostly news or research or OA resources but also included welcoming new members and responding to a member question. No posts were removed by the moderator. Administration of the platform, including managing license and enrolling new members, equated to approximately 0.5-1 hour/week, excluding initial setup and testing of the forum. Annual software license with educational institution discount was Aus $15 (US $11 normally Aus $100/year [US $71]).

### Psychological Determinants, Behaviors and Health Outcomes

The baseline and follow-up data for the psychological determinants, behaviors, and health outcomes are provided in Multimedia Appendices 4-6. The study was not powered for within- or between-group statistical analyses, and the large number of analyses increased the risk of type 1 errors (false positives). There is no evidence supporting a clinically meaningful change in any of the measures within the OSG group and no between-group differences that suggest which outcomes may be positively affected by OSG participation. A total of 8 participants from each of the control (8/22, 36%) and experimental (8/31, 26%) groups reported overall improvement because of the intervention or interventions. No participant reported an adverse event that could have been related to the intervention or interventions. The 2 outcomes with the largest between-group differences in change favoring OSG were self-efficacy for pain (between-group difference in change 0.5 [95% CI -0.4 to 1.4]) and the health literacy domain of *navigating health care services* (between-group difference in change 0.3 [95% CI 0.0-0.5]).

### Qualitative Evaluation

A total of 10 people participated in the interviews about their perspectives of participating in an OSG for their condition: 8 (80%) women, mean age 63.2 (SD 7.7) years, mean visits 8.4 (SD 5.5; range 3-18); 5 (50%) interviewees did not post at all; and the remaining 5 (50%) posted between 1 and 4 times. The interviews lasted between 15 and 35 minutes. Three main themes were derived from the interview transcripts: connections and support, information and advice, and barriers and facilitators. These themes are comprehensively described from the data elicited in the interviews; however, the analysis does not suggest equal importance of all inferences. In addition, we report the perspectives from 1 or 2 participants when they presented a new or disparate view, but these views may not necessarily be consistent with the views of the entire sample or the knee OA population.

Under the theme of connections and support, interviewees talked about the importance of *support* from others in managing the condition more generally, the *benefits* of connecting with other people with OA*,* and *preferring to spectate* rather than connect*.* In relation to *support, s*ome specifically mentioned the need for emotional support. Interviewees talked about how useful it was to be able to talk to people other than their family or friends and health care professionals:

Because it’s a consistent pain problem, having a place to go to when the pain is bad for other suggestions or information or even just some reassurance, which is not bothering your GP and it’s also not you complaining yet again to your family. Or maybe you don't want to [talk about it] because you don't want people in your workplace to realize it’s inhibiting your life, you don’t want them to think it’s going to inhibit what you can do in the workplace. [OSGs are] a neutral place to be able to go and I think that’s a real benefit and positive.

OSG10

Interviewees also talked about finding the *support* they received from health care professionals sometimes being inadequate, for example, because of the time constraints or costs of seeing health professionals:

I know they’ve got their 15 minutes or their half hour, whatever they’re charging for, but - doctors seem to be, in and out, thank you. They’re happy to prescribe something, but I don’t want to be prescribed anything.

OSG3

One interviewee said that they liked the idea of trying to *support* others by posting about their experiences on a forum. Finally, 1 interviewee said they felt “very supported” [OSG7] by the group moderator within the My Knee Community. It was not clear from the data whether these were disparate views or minor themes.

Some interviewees described *benefits* that they perceived they could gain from connecting with other people with OA. These included the reassurance that comes from knowing *you are not the only one*:

So I personally found it extremely helpful, and it made me realize that no, there are other people out there with the same if not worse conditions that are still doing other things.

OSG8

It’s just about sharing your experiences I suppose and helping to put your troubled mind at rest if that makes sense. I mean I didn’t have great expectations - it’s just a reassurance that mentally...And other people are in the same position.

OSG1

It was the personal comments that I really, I think, probably enjoyed the most...It’s hard to explain, I felt part of this, but nothing on a personal level. Just that it was a group that I was part of.

OSG6

A specific *benefit* of connecting with other people with knee OA through the OSG mentioned by some interviewees was motivation to keep up their self-management. They derived encouragement from reading about other people who were still active despite their pain or their age:

It’s encouraged me to be more active and to be a bit more proactive about my self-care.

OSG10

So being reminded about exercise, being reminded about the fact that those people were older and were doing a lot of exercise and pushing through their pain. Being reminded that being in pain doesn’t make any difference to–it was not going to injure me any further, it was not going to make things worse, that it would make things better. All of those sorts of things were useful.

OSG2

Many interviewees *preferred to spectate* rather than contribute to discussions or connect with others on the OSG:

I didn’t even post anything, I just read...Well, I didn’t really think I had anything to add.

OSG3

I probably always thought I would do nothing because that’s probably just my nature. I don’t post on Facebook. I just look.

OSG6

I enjoyed watching and listening and seeing what people were communicating on the chat, that was really interesting.

OSG10

Some had specific reasons for not posting, for example, they were not interested in social connections or relationships or they did not feel they fitted in:

I probably didn't use it as much as I thought I would. Because a lot of people were talking about a lot more problems and things like that and I couldn't really associate with what they were saying...I think it probably was more involved for the higher pain threshold.

OSG5

One interviewee perceived that the people in the OSG were older [OSG2] and therefore they did not feel they could be a part of the group. Another interviewee said they did not post because they were older:

I don’t post anywhere really. I guess because I’m older. I don’t know, just the privacy I suppose.

OSG6

Another interviewee was very clear that they did not want to connect with others, saying that they were not interested in emotional support or sympathy and just wanted to know how to *get rid of the pain* [OSG3]. Some interviewees suggested that they did not see the benefit in reading about other people’ experiences:

But people put up things that I’ve looked at, and gone oh yeah, well, good luck with that.

OSG1

In relation to the second theme of information and advice, interviewees talked about being *curious* about how others manage, wanting to *learn* about new management methods, and having *concern* about the accuracy of information being posted. *Curiosity* about what others were doing was the main reason for becoming a member of the OSG for some:

I like to know a little bit about everything. So whatever was new, I had a look at.

OSG3

People were also motivated to *learn* through sharing information about things that help and receive “people’s genuine opinions” [OSG6]:

I do have a belief that I can work on it myself. I can, you know, learn to cope with it and get it better again.

OSG7

Some interviewees made the point that they were looking for treatment ideas that they were not receiving from health professionals:

General advice because most of the health professionals, their answer is tablets or medications and stuff, and I’m a bit over that.

OSG4

Some interviewees said that people with knee OA often do not realize the other things they can do besides see health professionals, and meeting other people with the condition may be a way of learning about other strategies:

A lot of people sort of-how can I explain-they suffer in silence thinking, “All right, it’s just age,” and that sort of thing, and they don’t realize that there are opportunities there for them to get help to deal with it.

OSG8

Some of our interview participants found that hearing it from people with knee pain themselves encouraged them to try new things or be more active. Some mentioned they were managing a little bit better because of what they had learned:

I am now seeking a bit more attention for my knee. I've just been putting up with it and been resigned to this is the way it's going to be. So [the OSG] has prompted me to be more active.

OSG10

Alternatively, some were more interested in the advice from experts (ie, the moderator or new research findings). Interviewees appreciated the resources (eg, videos and exercise programs) that were introduced to the group and some reported finding that they had benefited from them:

By discovering those resources such as [painTRAINER]...that definitely helped me to find a way to manage my pain to my point that I don’t have any at the moment.

OSG7

In contrast, some interviewees said they did not learn anything because they already knew most of the information; there was nothing new for them.

A few interviewees expressed some *concern* that the things people were posting about may not be helpful or “recommended” [OSG7]. They did not want to get involved in the discussions, and this concern meant that they were less keen on the OSG:

I was just concerned that, you know, if they said, “Eat ants” other people might have gone and eaten ants because formic acid helps, or something [laughs]...There was someone who was sort of keeping an eye on it, but yes, to begin with I was just a bit concerned about, you know, “Whose advice do I follow?”

OSG7

The final theme was barriers and facilitators to engagement. *Barriers* included the perceived repetition within posts leading to much time reading. Conversely, the low level of activity on the site and infrequent new posts was also reported as contributing to reduced interest in engaging. One interviewee explained that they felt disappointed that they did not receive many replies to their post and that the conversations did not get going:

So even when you make a comment...you might not get a comment or any feedback.

OSG2

Several interviewees commented on difficulties with accessibility, particularly on mobile devices, although most interviewees thought that the web-based format was easy to access. In terms of *facilitators*, interviewees talked about the advantages of being on the web and asynchronous rather than in person, for example, liking the flexibility of being able to use it a lot or just a little:

And the fact that it is online and so you’re not having to physically go somewhere to go to a meeting, or to go a session, makes it easily portable and accessible for people in all sorts of ways and means.

OSG10

Some of the study participants set up notifications, and occasionally the moderator would push out a notification to remind people to visit. Both these strategies were mentioned as being helpful by some of our interviewees. In terms of suggestions to facilitate engagement, 1 interviewee would have liked a scheduled release of new information, so they would know when to go on and look at what was new. Several people suggested that a reminder or a prompt such as a weekly summary might help to encourage more engagement.

## Discussion

### Principal Findings

This pilot study found that a trial of the effect of an evidence-informed, expert-moderated, peer-to-peer online support group for people with knee OA (My Knee Community) is feasible in terms of study methods (recruitment, retention, and costs). Our findings suggest that future sample size calculations would need to allow for approximately 25% participant attrition given the 76% retention rate among our experimental group. However, our experimental intervention was not delivered to an acceptable extent as indicated by our measures of engagement and satisfaction. A total of 85% (35/41) of the participants adhered to the requirement to log on to the OSG at least once, which may seem to be an acceptable protocol adherence; however, given the overall lack of activity on the forum as well as the lack of activity by the study participant members, the dosage of any potentially important components would have been low. Only 55% (17/31) of the experimental group participants perceived benefit, and this was notably lower than for the control group’s perceived benefit from the information website alone. Overall satisfaction was 5.9/10, which is relatively low compared with satisfaction scores in other OSG studies [53,54]. In relation to the qualitative evaluation, our data suggest that most people with knee OA perceive that they need informational support and, to a much lesser extent, emotional support. However, the participants did not find the My Knee Community OSG particularly useful in meeting these needs. Some participants liked hearing about how other people manage, but in general, they were not willing to share their own experiences. In terms of perceived benefits, some participants found that the OA resources posted by the moderators were useful as this information was not normally provided by health professionals. The low level of activity meant that people quickly drifted away from the group. Facilitators of engagement included identifying as someone who fitted into the group and being comfortable with the technology. In relation to the impacts, the study was not powered to detect a difference in effect, and the dosage of intervention components experienced by participants was low. Of the numerous measures, self-efficacy for pain and the health literacy domain of *navigating health care services* are suggested as target outcomes in future trials.

As with our findings, previous studies have shown that posts in OSGs can mostly be categorized as being supportive or informational [55,56]. Emotional support, positive feedback from others, and reinforcement of decisions was found to occur in other OSGs [55-58]. Unfortunately, there was little such peer-to-peer interaction in the My Knee Community. Nonetheless, our OSG helped some people to feel reassured and more motivated to actively self-manage. In relation to information, we found that some people favored the information from other people with knee OA, whereas others had a clear preference for information from the *experts*. Our earlier survey study indicated that trustworthiness of the organizing group and incorporating health professionals or expert peer leaders would be important to potential members [14], and this was substantiated by our findings. During the study period in our OSG, the most popular topics were related to exercise, followed by other treatment options, such as weight loss and supplements. Other studies have shown that people most often talk about medications and symptom management [12,59]. Differences may be due to the type of informational posts provided by the My Knee Community moderators.

It was clear from our study that most people prefer to be passive members rather than actively post and engage with other members. Despite this, our qualitative data indicate that many people know they need support and know about the benefits of connecting with others. Considerable evidence from the field of positive psychology shows the importance of talking to other people, including the value of talking to strangers [60]. Research shows that people often prefer not to connect with others, but they are happier if they do [60]. Perceived fear about conversation enjoyment and pessimism about how they will be perceived are typical barriers [60]. People who are more engaged in OSGs may experience greater gains in health literacy and self-esteem than those who post infrequently or only *lurk* [54,61]. Thus, efforts to promote the OSG as a safe place to share and express their feelings, respond positively to posts, and role model suitable posts may be warranted to encourage activity. On the other hand, research has shown that people can benefit from OSGs even if they avoid posting [54]. Posting behavior may be largely determined by personality traits and the nature of the condition (not life-threatening and relatively common in the community). These factors may influence both engagement and satisfaction with peer-to-peer OSGs for OA.

Our quantitative data indicated that self-efficacy and health literacy may be mechanisms by which OSGs can lead to improved health outcomes. This is supported by findings from previous studies, which suggest that the sharing of experiences in chronic condition on web-based communities helps improve health literacy and the quality of self-management plans [57,58]. In addition, multiple cohorts and observational studies of people with nonmusculoskeletal disorders have reported significant positive effects on self-efficacy following participation in OSGs [62-65]. Both health literacy and self-efficacy are considered foundational for effective chronic disease self-management [56,58,66,67]. Our qualitative data additionally suggest that people may benefit through increased motivation to actively self-manage (ie, feeling encouraged by other posts) and perhaps also simply through boosting their mood. The potential mechanisms require further investigation.

Our feasibility study has led us to consider some important modifications to our OSG before continuing to a full-scale intervention study. First, a critical mass of members may be needed in an OSG for knee OA to create an atmosphere that retains and benefits members. People tended to be reluctant to post, but at the same time, lack of activity was given as a reason to disengage. A large membership may mean that enough people become regular posters to maintain momentum and consequently the interest of the wider group. A larger membership might also lead to greater diversity of member characteristics and diversity of opinion [14], which might, in turn, lead to wider appeal. Second, we discovered that many of our study participants seemed to be motivated by curiosity or had a preconceived expectation of what they wanted to find out from the community. Both types were quick to disengage. Therefore, we recommend that expectations are clarified before people join by explaining that the My Knee Community is a forum for sharing experiences, discussing, and supporting each other (not just a knee OA information resource). For trial purposes, we recommend focusing recruitment on people who are interested in sharing and supporting. As noted in a review on mechanisms of action in group-based health behavior change interventions, groups are not for everyone. Participant selection and matching are an important preliminarily part of setting up effective groups, including effective online groups [15]. We found that inclusion of the moderator team was helpful for safety, responding to some types of questions, and for communicating trustworthy information and therefore recommend that this feature remains. This recommendation is consistent with our earlier survey study, which concluded that a moderator role is important for explaining complex topics and maintaining trustworthiness [14]. That study also highlighted the need for people to enjoy participating in the forum to maintain participation [14]. Therefore, we will consider some additional strategies to increase enjoyment and ongoing interest, for example, adding humor, changing the design to be more attractive, emailing weekly summaries, sending reminders or other notifications (but not too many), and improving accessibility (mobile phone interface) and ease of log-in.

### Limitations

This pilot study was not designed to evaluate impacts, and our findings are limited by the small sample sizes for both quantitative and qualitative analysis (only 10 out of 41 participants agreed to be interviewed) and the uncertainty about the dosage of the intervention that was delivered. These sources of bias are likely to underestimate the impact. Other limitations include error and noise inherent in the self-reported measures [68] and potential sampling bias because of our recruitment predominantly using social media. Both our strategies for recruiting My Knee Community members and study participants may not have reached the people most likely to benefit from an OSG, for example, people who are isolated or lack social support for other reasons and people who have lowest health literacy and access to health professionals and high value health care. The limitations to generalizability of the findings include the digital literacy requirements, inclusion based on self-diagnosis of knee OA according to clinical presentation, and the Australian health care context.

### Conclusions

Future research should consider cost as well as health benefits. Health benefits are likely to be small at best; however, because of the large number of people with knee OA and the relatively low cost and safety of OSG interventions, OSGs for OA may have value as part of a range of options. Importantly, we recommend that the intervention be delivered only to those inclined to engage with the format. Our study found a full trial of an expert-moderated, peer-to-peer online support group intervention to be feasible provided the OSG can engage members and facilitate active participation. OSGs are rapidly growing in popularity and may provide a range of benefits for several health conditions or chronic disease–related problems. This study contributes to evidence-informed implementation and use of OSGs for improving self-management behaviors and health outcomes for people with knee OA as well as future trial design.

## Appendix

Multimedia Appendix 1 The evaluation plan related to study feasibility.

Multimedia Appendix 2 The evaluation plan related to the intervention impact mapped to the logic model components.

Multimedia Appendix 3 The qualitative interview guide.

Multimedia Appendix 4 Psychological determinants: summary measures and estimated mean differences in change (95% CI).

Multimedia Appendix 5 Behaviors: summary measures and estimated mean differences in change (95% CI).

Multimedia Appendix 6 Health outcomes: summary measures and estimated mean differences in change (95% CI).

Multimedia Appendix 7 CONSORT-EHEALTH (V 1.6.1) - Submission_Publication Form.

## Abbreviations

NRS: numerical rating scale
OA: osteoarthritis
OSG: online support group
REDCap: Research Electronic Data Capture
