# Supplementary material for: Expert-Moderated Peer-to-Peer Online Support Group for People With Knee Osteoarthritis: Mixed Methods Randomized Controlled Pilot and Feasibility Study
Source: JMIR Form Res. 2022 Jan 17;6(1):e32627. doi: 10.2196/32627 (PMC8804962; doi:10.2196/32627)
Supplement: Multimedia Appendix 1 [file formative_v6i1e32627_app1.pdf]

Multimedia Appendix A. The evaluation plan related to feasibility.

Item	Method	Time points
Experimental intervention (online support group) feasibility		
Perceived benefit:		
	My Joint Pain - Do you think you benefitted from using the My Joint Pain website? (Y/N)	3 months
	My Knee Community - Do you think you benefitted from using the My Knee Community? (Y/N)	3 months
Satisfaction:		
	My Knee Community (0-10 NRS):<br>Please rate how satisfied you are with the following in relation to the My Knee Community (Not at all satisfied - completely satisfied):<br>– Overall<br>– Quality of advice/information<br>– Amount of information<br>– Ease of use<br>– Relationships developed with other participants<br>– Input from the expert moderator	3 months
Engagement:		
	Self-report engagement<br>– How many times did you visit the OSG over the past 3 months? (Never, 1-2 times, 3-5 times, 6-10 times, >10)<br>– How often did you read discussion board posts? (Never or rarely, once every two or three weeks, once or twice per week, more than twice per week)<br>– How often did you post on the discussion board? (Never or rarely, once every two or three weeks, once or twice per week, more than twice per week)	3 months
	Software analysis:<br>– Proportion of participants accessing My Knee Community at least once<br>– Number of times visited by study participants<br>– Number of topics viewed by study participants<br>– Number of posts read by study participants<br>– Number of posts created by study participants<br>– Posts with most reads (reported narratively)<br>– Posts with most likes (reported narratively)<br>– Number of participants categorised as ‘posters’ vs ‘lurkers’ vs ‘no participation’	End of trial
Study feasibility		
Cost		
	– Moderator activity (number and type of posts)<br>– Administration time<br>– Software licence cost	End of trial
Participant recruitment		
	– Recruitment sources (paid, unpaid)<br>– Recruitment costs per participant<br>– Recruitment rate (number/week)	End of trial
Participant retention	Proportion of participants completing 3-month assessment from each group	End of trial
