# Supplementary material for: Expert-Moderated Peer-to-Peer Online Support Group for People With Knee Osteoarthritis: Mixed Methods Randomized Controlled Pilot and Feasibility Study
Source: JMIR Form Res. 2022 Jan 17;6(1):e32627. doi: 10.2196/32627 (PMC8804962; doi:10.2196/32627)
Supplement: Multimedia Appendix 2 [file formative_v6i1e32627_app2.pdf]

Multimedia Appendix 2. The evaluation plan related to intervention impact mapped to the logic model components.

Item	Method	Time points
**Inputs - Participants**		
Gender	Male or female or other	Baseline
Age	Years	Baseline
Location	Major city/inner regional/outer regional/remote/very remote	Baseline
Educational level	Self-reported highest level of education completed	Baseline
Current employment status	Self-reported current employment status (employed full time or part time/casual, or not in paid employment)	Baseline
Duration of knee pain symptoms	Range from 'Less than one year' to 'More than 10 years'	Baseline
Pain in other parts of the body	List of body parts, yes/no	Baseline
Duration since first visit to doctor for knee pain	Range from 'Less than one year ago' to 'More than 10 years ago'	Baseline
Prior experience with OSG, other support groups or self-management programs	Yes/No	Baseline
**Outcomes - Psychological determinants**		
Motivation (intention) for core behaviours	0-10 NRS:<br>- Please rate how important it is for you to be more active / exercise / lose weight (Not at all important - Very important)<br>- How much do you agree with the following statement? I intend to increase my activity / exercise / lose weight? (Not at all - Completely agree)<br>- How motivated are you to be more active / exercise / lose weight? (Not at all motivated - Very motivated)	Baseline, 3 months
Attitudes toward self-management (activation)	Patient Activation Measure (13 items, 0-100)	Baseline, 3 months
Self-efficacy	Arthritis Self-Efficacy Scale<br>- Pain subscale (5 items, 1-10)<br>- Function subscale (3 items, 1-10)<br>- Other symptoms subscale (6 items, 1-10)	Baseline, 3 months
Impact of health education	Health Education Impact Questionnaire (40 items, 8 domains, 1-4)<br>- Health Directed Behaviour<br>- Positive and Active Engagement in Life<br>- Self-monitoring and Insight<br>- Constructive Attitudes and Approaches<br>- Skill and Technique Acquisition<br>- Social Integration and Support<br>- Health Services Navigation<br>- Emotional Distress	Baseline, 3 months

Item	Method	Time points
Health literacy	Health Literacy Questionnaire<br>- Feeling understood and supported (4 items, 0-4)<br>- Having sufficient information (4 items, 0-4)<br>- Actively managing my health (4 items, 0-4)<br>- Social support for health (4 items, 0-4)<br>- Appraisal of health information (4 items, 0-4)<br>- Ability to actively engage (4 items, 0-5)<br>- Navigating the healthcare system (4 items, 0-5)<br>- Ability to find good health information (4 items, 0-5)<br>- Understand health information (4 items, 0-5)	Baseline, 3 months
Coping	Brief Coping Strategy Questionnaire (14 items, 7 domains, 0-6)<br>- Diverting attention<br>- Reinterpreting pain sensations<br>- Coping self statements<br>- Ignoring sensations<br>- Praying hoping<br>- Catastrophising<br>- Increase behavioural activities	Baseline, 3 months
Perceived social support	Duke-UNC Functional Social Support Questionnaire (8 items, 1-5)	Baseline, 3 months
Fear of movement (kinesiophobia)	Brief Fear of Movement Scale for Osteoarthritis (6 items, 0-20)	Baseline, 3 months
**Outcomes - Self-management behaviours**		
Physical activity	Incidental and Planned Exercise Questionnaire - past week (hours/week)	Baseline, 3 months
Physical activity	- How many days in the past week did you do 30 minutes of moderate intensity physical activity? *Moderate intensity* means being active at a level that makes you feel at least a little out of breath or feels 'somewhat hard' or harder (0-7)	Baseline, 3 months
Strengthening exercise behaviour	- How many days in the past week did you do leg strengthening exercises? (0-7)	Baseline, 3 months
Weight loss behaviour	0-10 NRS:<br>- If you need to lose weight, how much effort are you currently making? (no effort – maximal effort)	Baseline, 3 months
**Outcomes - Health outcomes and healthcare usage**		
Global rating of change	7-point Likert scale:<br>- Overall change in knee condition since commencing in the study (Much worse - Much better). Improved: ≥ 5	3 months
Overall average knee pain	0-10 NRS:<br>- Please rate your overall average knee pain in the last week (No pain - Worst pain possible)	Baseline, 3 months
Activity-related pain	0-10 NRS:<br>- During the past week, what was the worst pain you felt during an activity that usually brings on your knee pain (No pain - Worst pain imaginable)	Baseline, 3 months

Item	Method	Time points
Physical function	Western Ontario & McMaster University Osteoarthritis Index (WOMAC) Physical Function subscale (17 items, 0-68, higher scores indicate worse function)	Baseline, 3 months
Sleep	0-10 NRS:<br>- In the past 7 days, how would you rate your sleep quality overall? (Terrible – Excellent)	Baseline, 3 months
Fatigue	0-10 NRS:<br>- How fatigued do you currently feel? (Not at all fatigued-Extremely fatigued)	Baseline, 3 months
Mood (depressive symptoms)	Patient Health Questionnaire (9 items, 0-27)	Baseline, 3 months
Health-related quality of life	Assessment of Quality of Life Instrument (20 items, -0.04 to 1.00, 1.00 equates to highest quality of life)	Baseline, 3 months
**Outcomes - Harms**		
Related adverse events	- Have you had any new health problems or symptoms, or have any of your existing health conditions or symptoms worsened since you started in the study? (Number and type)	3 months
