# Supplementary material for: Expert-Moderated Peer-to-Peer Online Support Group for People With Knee Osteoarthritis: Mixed Methods Randomized Controlled Pilot and Feasibility Study
Source: JMIR Form Res. 2022 Jan 17;6(1):e32627. doi: 10.2196/32627 (PMC8804962; doi:10.2196/32627)
Supplement: Multimedia Appendix 3 [file formative_v6i1e32627_app3.pdf]

Multimedia Appendix 3. Qualitative interview guide.

Consent reviewed prior to the interview
Preamble: We are interested in finding out about your experiences with the online support group and your thoughts on those experiences. There are no right or wrong answers. We are very keen to hear your opinions or reactions, positive or negative or neutral. Please stop me at any time if you need to take a break, or want to end the interview for any reason. Also, you don't have to answer every question – just let me know if you want to move on from something. Before I start, do I have your permission to audio-record this session?

1. Can tell me about your knee pain?
Possible prompts
a) how long have you had it?
b) what do you think is causing it?
c) what things have you done about, for example, have you been to see someone for treatment or tried anything to help relieve pain?

2. What did you expect to get from the online support group when you first signed up for the study?
Possible prompts
a) Why did you join?
b) How did you think it would help?
c) What did you think you would do online?
d) How (if at all) was your experience different to what you expected?
e) Do you think you got from it what you had hoped to get?
f) Can you go into a little more detail about that?

3. Please describe how you used the online support group?
Possible prompts
a) What sorts of things did you read on the discussion forum?
b) Can you describe how it felt to read the posts of others?
c) Did you read anything that you disagreed with? What did you do?
d) What was your experience posting on the online support group?
If they didn't post anything – What were your reasons for not posting on the OSG?
e) Did you feel you formed any relationships with the other members or the moderator?
f) Can you go into a little more detail about that?

4. What do you think are the benefits for people of participating in online support groups such as this one?
Possible prompts
a) How do you think it might help them? Did you personally experience any benefits?
b) Has the OSG led you to make any changes?
c) Can you go into a little more detail about that?

5. What sorts of things made it difficult for you to use the OSG?
Possible prompts

a) For example, were there any difficulties with technical aspects or finding what you were looking for?
b) Can you go into a little more detail about that?

6. What sorts of things made it easier for you use the OSG?
Possible prompts
a) What made it easy to use?
b) Did you find it easy to remember to go online?
c) Can you go into a little more detail about that?

7. (Based on your experiences in the study) would you consider using an OSG in the future?
Possible prompts
a) Why/why not?
b) how it could be changed/improved in the future to entice you/others to participate?

8. Is there anything else you would like to mention that we haven't discussed today?
Additional question A. What sort of support do you think people with knee OA need?
Possible prompts
a) Do you think people with knee OA need support from other people or from health professionals
b) Can you go into a little more detail about that?
c) Why do you think this?
d) Have you ever felt you needed support?
e) Can you explain a bit more about your feelings about the importance of being able to access support when you need it?

Additional question B. How do you think people with knee OA normally get the support they need?
Possible prompts
a) What things have you done or tried?
b) How important do you think it is for people with knee OA to be supported?
