# Supplementary material for: Expert-Moderated Peer-to-Peer Online Support Group for People With Knee Osteoarthritis: Mixed Methods Randomized Controlled Pilot and Feasibility Study
Source: JMIR Form Res. 2022 Jan 17;6(1):e32627. doi: 10.2196/32627 (PMC8804962; doi:10.2196/32627)
Supplement: Multimedia Appendix 4 [file formative_v6i1e32627_app4.pdf]

Multimedia Appendix 4. Psychological determinants.

Change within groups, and difference in change between groups (adjusted for baseline value of outcome). Change within groups is follow-up minus baseline. Between group difference is change in experimental group (online support group) minus change in control group. Data are mean (SD) [95% confidence interval].

	Control			Online Support Group			Between group difference in change
	Baseline (n=22)	Follow-up (n=22)	Within-group change (n=22)	Baseline (n=41)	Follow-up (n=31)	Within-group change (n=31)	(Adjusted for baseline values)
**Motivation:**							
How important do you think it is for people with knee OA to be physically active? 0-10[a]	8.5 (1.5)	9.0 (1.4)	0.5 (1.4)	8.6 (1.6)	8.4 (1.5)	-0.3 (1.4)	-0.7 [-1.4, 0.0]
How much do you agree with the statement: I intend to increase my physical activity? 0-10[a]	7.3 (2.6)	8.2 (1.9)	1.0 (2.2)	8.0 (1.7)	7.0 (2.2)	-0.9 (2.3)	-1.5 [-2.5, -0.4]
How motivated are you to be more physically active than currently? 0-10[a]	7.0 (2.7)	7.9 (1.9)	0.9 (2.7)	7.5 (2.2)	6.8 (2.2)	-0.6 (1.8)	-1.3 [-2.3, -0.2]
How important do you think it is for people with osteoarthritis to keep their weight down? 0-10[a]	9.3 (0.9)	9.3 (1.2)	0.0 (1.0)	9.3 (0.9)	8.6 (1.5)	-0.7 (1.2)	-0.7 [-1.3, 0.0]
How much do you agree with the statement: I intend to try and lose some weight? 0-10[a,b]	8.3 (3.8)	7.9 (2.8)	-0.3 (1.9)	8.4 (2.9)	8.2 (2.1)	-0.3 (1.8)	0.2 [-0.8, 1.2]
How motivated are you to take some actions to lose some weight? 0-10[a,b]	7.0 (3.4)	6.7 (2.9)	-0.1 (2.7)	7.7 (2.9)	7.0 (2.4)	-0.6 (2.6)	0.1 [-1.3, 1.5]
**Self-efficacy:**							
ASES Pain (5 items, 1-10)[c]	5.3 (1.6)	5.9 (1.6)	0.6 (1.6)	5.8 (1.9)	6.6 (1.8)	0.7 (2.1)	0.5 [-0.4, 1.4]
ASES Function (3 items, 1-10)[c]	5.9 (2.5)	6.8 (2.5)	0.9 (1.6)	5.8 (2.1)	7.2 (2.1)	1.1 (1.8)	0.3 [-0.6, 1.2]
ASES Other symptoms (6 items, 1-10)[c]	5.9 (1.6)	6.8 (1.5)	0.9 (1.5)	6.0 (1.9)	6.7 (1.6)	0.5 (1.6)	-0.3 [-1.0, 0.5]

	Control			Online Support Group			Between group difference in change
	Baseline (n=22)	Follow-up (n=22)	Within-group change (n=22)	Baseline (n=41)	Follow-up (n=31)	Within-group change (n=31)	(Adjusted for baseline values)
**Activation to self-manage:**							
Patient Activation Measure 0-100[a]	71.0 (12.1)	77.4 (11.7)	6.4 (11.6)	71.9 (15.7)	79.5 (12.9)	7.6 (14.4)	1.7 [-4.4, 7.9]
**Health Education Impact Questionnaire:**							
Health directed behaviour[a]	2.9 (0.7)	3.0 (0.7)	0.1 (0.8)	2.8 (0.7)	3.0 (0.6)	0.1 (0.7)	0.0 [-0.4, 0.3]
Positive and active engagement in life[a]	3.1 (0.5)	3.2 (0.6)	0.1 (0.6)	3.0 (0.5)	3.1 (0.5)	0.0 (0.5)	-0.1 [-0.4, 0.1]
Self-monitoring and insight[a]	2.9 (0.4)	3.1 (0.3)	0.1 (0.4)	2.9 (0.4)	3.1 (0.3)	0.2 (0.5)	0.0 [-0.1, 0.2]
Constructive attitudes and approaches[a]	3.0 (0.4)	3.1 (0.4)	0.1 (0.5)	3.1 (0.5)	3.2 (0.4)	0.1 (0.5)	0.1 [-0.2, 0.3]
Skill and technique acquisition[a]	2.6 (0.5)	3.0 (0.4)	0.4 (0.5)	2.6 (0.5)	3.0 (0.5)	0.3 (0.5)	0.0 [-0.3, 0.2]
Social integration and support[a]	2.6 (0.5)	2.8 (0.6)	0.2 (0.4)	2.5 (0.6)	2.7 (0.6)	0.2 (0.5)	-0.1 [-0.3, 0.2]
Health services navigation[a]	3.1 (0.5)	3.1 (0.5)	0.0 (0.5)	3.0 (0.5)	3.2 (0.3)	0.1 (0.5)	0.1 [-0.1, 0.3]
Emotional distress[c]	2.1 (0.5)	2.0 (0.5)	-0.2 (0.5)	2.1 (0.6)	2.0 (0.5)	-0.1 (0.6)	0.1 [-0.2, 0.3]
**Health literacy (Health Literacy Questionnaire):**							
Feeling understood and supported (0-4)[a]	2.9 (0.5)	3.1 (0.5)	0.2 (0.4)	3.0 (0.6)	3.3 (0.4)	0.2 (0.5)	0.1 [-0.1, 0.3]
Having sufficient information (0-4)[a]	2.6 (0.4)	3.0 (0.4)	0.4 (0.3)	2.7 (0.6)	3.0 (0.4)	0.4 (0.4)	0.0 [-0.2, 0.2]
Actively managing my health (0-4)[a]	2.8 (0.5)	2.9 (0.5)	0.0 (0.3)	2.8 (0.6)	2.9 (0.4)	0.2 (0.4)	0.1 [-0.1, 0.3]
Social support for health (0-4)[a]	2.6 (0.5)	2.8 (0.5)	0.3 (0.3)	2.6 (0.7)	2.7 (0.7)	0.1 (0.5)	-0.1 [-0.3, 0.1]
Appraisal of health information (0-4)[a]	2.9 (0.5)	3.1 (0.4)	0.2 (0.4)	3.0 (0.5)	3.2 (0.4)	0.2 (0.4)	0.1 [-0.1, 0.3]
Ability to actively engage (0-5)[a]	3.5 (0.8)	3.8 (0.6)	0.3 (0.6)	3.7 (0.8)	4.0 (0.6)	0.2 (0.5)	0.1 [-0.2, 0.3]
Navigating the healthcare system (0-5)[a]	3.4 (0.5)	3.6 (0.6)	0.2 (0.4)	3.5 (0.7)	4.0 (0.6)	0.4 (0.5)	0.3 [0.0, 0.5]

	Control			Online Support Group			Between group difference in change
	Baseline (n=22)	Follow-up (n=22)	Within-group change (n=22)	Baseline (n=41)	Follow-up (n=31)	Within-group change (n=31)	(Adjusted for baseline values)
Ability to find good health information (0-5)[a]	3.7 (0.4)	4.1 (0.4)	0.3 (0.5)	3.8 (0.6)	4.1 (0.6)	0.2 (0.5)	-0.1 [-0.3, 0.2]
Understand health information (0-5)[a]	4.1 (0.4)	4.2 (0.4)	0.1 (0.4)	4.1 (0.6)	4.2 (0.6)	0.1 (0.4)	0.0 [-0.2, 0.3]
**Coping (Brief Coping Strategies Questionnaire, all 0-6):**							
Diverting attention[a]	1.9 (1.7)	2.4 (1.4)	0.5 (1.6)	2.1 (1.4)	2.8 (1.8)	0.6 (1.2)	0.2 [-0.5, 0.9]
Reinterpreting pain sensations[a]	1.5 (1.7)	1.8 (1.5)	0.2 (1.1)	1.4 (1.2)	2.0 (1.4)	0.6 (1.4)	0.3 [-0.3, 1.0]
Coping self statements[a]	3.2 (1.8)	3.3 (1.3)	0.1 (1.6)	3.3 (1.0)	3.6 (1.2)	0.2 (0.9)	0.2 [-0.4, 0.8]
Ignoring sensations[a]	2.7 (2.1)	3.2 (1.6)	0.5 (1.8)	2.5 (1.2)	2.8 (1.1)	0.2 (1.2)	-0.3 [-1.0, 0.3]
Praying hoping[a]	1.5 (1.7)	1.7 (1.7)	0.2 (1.2)	1.7 (1.3)	1.4 (1.3)	-0.2 (1.2)	-0.4 [-1.0, 0.3]
Catastrophising[c]	1.5 (1.6)	1.2 (1.1)	-0.3 (1.3)	1.9 (1.4)	1.5 (1.0)	-0.3 (1.2)	0.1 [-0.4, 0.7]
Increase behavioural activities[a]	2.8 (1.4)	3.3 (1.4)	0.5 (1.6)	2.9 (1.3)	3.2 (1.3)	0.2 (0.8)	-0.2 [-0.8, 0.4]
**Other:**							
Social support (Duke-UNC Functional Social Support Questionnaire, 8 items, 1-5)[a]	3.8 (1.0)	3.9 (1.1)	0.1 (0.7)	3.6 (1.1)	3.8 (1.2)	0.2 (0.8)	0.0 [-0.4, 0.4]
Kinesiophobia (Brief Fear of Movement Scale for Osteoarthritis, 0-20)[c]	11.9 (3.3)	12.4 (3.1)	0.5 (2.6)	14.1 (3.8)	12.6 (3.7)	-1.3 (3.1)	-1.2 [-2.7, 0.4]

[a] For change within groups, positive change indicates improvement. For difference in change between groups, positive difference favours online support group.

[b] Data for those selecting ‘not applicable’ excluded

[c] For change within groups, negative change indicates improvement. For difference in change between groups, negative difference favours online support group.

ASES = Arthritis Self-Efficacy Scale
