# Supplementary material for: Expert-Moderated Peer-to-Peer Online Support Group for People With Knee Osteoarthritis: Mixed Methods Randomized Controlled Pilot and Feasibility Study
Source: JMIR Form Res. 2022 Jan 17;6(1):e32627. doi: 10.2196/32627 (PMC8804962; doi:10.2196/32627)
Supplement: Multimedia Appendix 5 [file formative_v6i1e32627_app5.pdf]

Multimedia Appendix 5. Behaviors.

Change within groups, and difference in change between groups (adjusted for baseline value of outcome). Change within groups is follow-up minus baseline. Between group difference is change in experimental group (online support group) minus change in control group. Data are mean (SD) [95% confidence interval].

	**Control**			**Online Support Group**			**Between group difference in change**
	Baseline (n=22)	Follow-up (n=22)	Within-group change (n=22)	Baseline (n=41)	Follow-up (n=31)	Within-group change (n=31)	(Adjusted for baseline values)
Physical activity, hours/week[a]	26.2 (14.8)	26.1 (17.4)	-0.1 (11.1)	26.6 (21.2)	24.8 (16.0)	-2.9 (22.7)	-1.8 [-10.2, 6.5]
Days in the past week of 30 min moderate physical activity, 0-7 days[a]	3.2 (2.0)	3.5 (2.3)	0.3 (1.4)	3.2 (1.9)	3.7 (1.6)	0.3 (1.6)	0.0 [-0.7, 0.8]
Days in the past week of leg strengthening exercises, 0-7 days[a]	3.1 (2.5)	3.0 (2.1)	-0.1 (2.2)	2.3 (2.2)	2.5 (1.7)	0.3 (2.0)	-0.1 [-1.1, 0.8]
If you need to lose weight, how much effort are you currently making? 0-10[a,b]	5.1 (2.9)	5.3 (2.2)	0.3 (2.7)	5.9 (3.0)	5.9 (2.1)	0.1 (2.3)	0.3 [-0.9, 1.6]

[a] For change within groups, positive change indicates improvement. For difference in change between groups, positive difference favours online support group.

[b] Data for those selecting ‘not applicable’ excluded.
