# Supplementary material for: Expert-Moderated Peer-to-Peer Online Support Group for People With Knee Osteoarthritis: Mixed Methods Randomized Controlled Pilot and Feasibility Study
Source: JMIR Form Res. 2022 Jan 17;6(1):e32627. doi: 10.2196/32627 (PMC8804962; doi:10.2196/32627)
Supplement: Multimedia Appendix 6 [file formative_v6i1e32627_app6.pdf]

Multimedia Appendix 6. Health outcomes.

Change within groups, and difference in change between groups (adjusted for baseline value of outcome). Change within groups is follow-up minus baseline. Between group difference is change in experimental group (online support group) minus change in control group. Data are mean (SD) [95% confidence interval] unless otherwise stated.

	Control			Online Support Group			Between group difference in change
	Baseline (n=22)	Follow-up (n=22)	Within-group change (n=22)	Baseline (n=41)	Follow-up (n=31)	Within-group change (n=31)	(Adjusted for baseline values)
Global change overall, n (%) improved[a]		8 (36%)			8 (26%)		
Average knee pain in past week, 0-10[b]	5.4 (2.3)	4.1 (1.9)	-1.2 (2.8)	5.8 (1.8)	4.3 (2.2)	-1.2 (2.3)	0.1 [-1.0, 1.2]
Worst knee pain in past week, 0-10[b]	6.8 (1.9)	5.8 (2.2)	-1.0 (2.2)	6.9 (2.0)	5.4 (2.3)	-1.2 (2.4)	-0.3 [-1.5, 0.9]
WOMAC (physical function subscale), 0-68[b]	23.6 (11.0)	18.5 (11.8)	-5.0 (8.4)	26.5 (11.9)	19.9 (11.3)	-5.1 (9.6)	0.4 [-4.5, 5.2]
Sleep quality in the past week, 0-10[c]	5.5 (2.0)	6.0 (1.5)	0.5 (1.9)	5.2 (2.2)	5.8 (2.5)	0.4 (2.1)	-0.2 [-1.1, 0.9]
Fatigue, 0-10[b]	4.5 (2.7)	4.5 (2.6)	0.0 (3.0)	5.1 (2.2)	5.6 (2.3)	0.8 (3.0)	1.1 [-0.3, 2.4]
Depressive symptoms (PHQ-9) 0-27[b]	6.0 (6.3)	4.0 (3.3)	-2.0 (4.3)	6.6 (5.5)	6.1 (4.8)	-0.1 (3.0)	2.0 [0.5, 3.5]
Quality of life (AQoL), -0.04 to 1.00[c]	0.63 (0.23)	0.73 (0.16)	0.10 (0.11)	0.63 (0.18)	0.70 (0.15)	0.05 (0.11)	-0.04 [-0.10, 0.01]

[a] Between group difference is odds ratio [95% confidence interval] of improved global rating of change if in the experimental group.

[b] For change within groups, negative change indicates improvement. For difference in change between groups, negative difference favours online support group.

[c] For change within groups, positive change indicates improvement. For difference in change between groups, positive difference favours online support group.

WOMAC = Western Ontario and McMaster Universities Osteoarthritis Index

PHQ-9 = 9-item Patient Health Questionnaire

AQoL = Assessment of Quality of Life instrument
